# Bis(2-amino-6-methyl­pyridinium) tetra­chloridocuprate(II)

**DOI:** 10.1107/S160053680904923X

**Published:** 2009-11-21

**Authors:** Jiang Gong, Gang Chen, Shi-Feng Ni, Yong-Yao Zhang, Hai-Bin Wang

**Affiliations:** a Department of Medicine, Tibet Nationalities Institute, Xianyang, Shaanxi 712082, People’s Republic of China; b Key Laboratory of Resource Biology and Biotechnology in Western China, Ministry of Education, College of Life Science, Northwest University, Xi’an 710069, People’s Republic of China; c College of Pharmaceutical Sciences, Zhejiang University of Technology, Hangzhou 310014, People’s Republic of China; d College of Chemical Engineering and Materials Science, Zhejiang University of Technology, Hangzhou 310014, People’s Republic of China

## Abstract

The title compound, (C_6_H_9_N_2_)_2_[CuCl_4_], contains a distorted tetra­hedral [CuCl_4_]^2−^ anion and two protonated amino­pyridinium cations. The geometries of the protonated amino­pyridinium cations reveal amine–imine tautomerism. The crystal packing is influenced by N—H⋯Cl and C—H⋯Cl hydrogen bonds and π–π stacking inter­actions [centroid–centroid distances = 3.635 (4) and 3.642 (4)°].

## Related literature

For a series of compounds with formula A_2_[*MX*_4_], where *A* is an organic cation, usually a protonted base, *M* is a divalent transition metal ion and *X* is a halide (Cl, Br), see: Hammar *et al.* (1997 ▶). For complexes in which *A* is a protonated alkyl­amine, see: Zhou & Drumheller (1990 ▶), a heterocycle such as pyridine, see: Place & Willett (1987 ▶), 2-amino­pyrimidine, see: Zanchini & Willett (1990 ▶) and 2-amino-3-methyl­pridine, see: Coffey *et al.* (2000 ▶). For bond lengths and angles in related structures, see: Antolini *et al.* (1988 ▶); Zhang *et al.* (2005 ▶); Jin, Shun *et al.* (2005 ▶); Feng *et al.* (2007 ▶); Nahringbauer & Kvick (1977 ▶). For other 2-amino­pyridinium structures, see: Luque *et al.* (1997 ▶); Jin *et al.* (2000 ▶, 2001 ▶); Jin, Tu *et al.* (2005 ▶). For studies on the tautomeric forms of 2-aminopyridine systems, see: Inuzuka & Fujimoto (1986 ▶, 1990 ▶); Ishikawa *et al.* (2002 ▶).
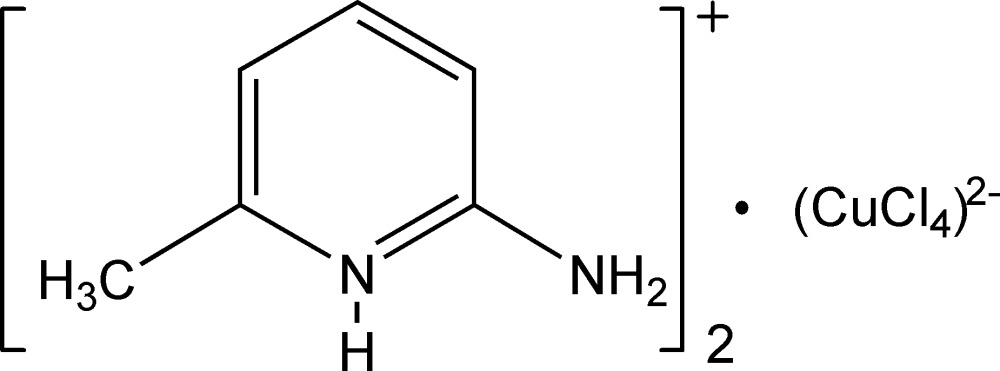


## Experimental

### 

#### Crystal data

(C_6_H_9_N_2_)_2_[CuCl_4_]
*M* *_r_* = 423.65
Triclinic, 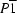

*a* = 7.7466 (17) Å
*b* = 8.0372 (18) Å
*c* = 14.969 (3) Å
α = 78.922 (4)°
β = 82.154 (4)°
γ = 89.911 (4)°
*V* = 905.8 (3) Å^3^
*Z* = 2
Mo *K*α radiation
μ = 1.79 mm^−1^
*T* = 273 K
0.35 × 0.34 × 0.30 mm

#### Data collection

Bruker SMART APEX area-detector diffractometer
Absorption correction: multi-scan (*SADABS*; Bruker, 2000 ▶) *T*_min_ = 0.549, *T*_max_ = 0.578
4783 measured reflections
3161 independent reflections
2874 reflections with *I* > 2σ(*I*)
*R*_int_ = 0.013

#### Refinement

*R*[*F* ^2^ > 2σ(*F* ^2^)] = 0.034
*wR*(*F* ^2^) = 0.090
*S* = 1.05
3161 reflections
190 parameters
H-atom parameters constrained
Δρ_max_ = 0.55 e Å^−3^
Δρ_min_ = −0.33 e Å^−3^

### 

Data collection: *SMART* (Bruker, 2000 ▶); cell refinement: *SAINT* (Bruker, 2000 ▶); data reduction: *SAINT*; program(s) used to solve structure: *SHELXTL* (Sheldrick, 2008 ▶); program(s) used to refine structure: *SHELXL97* (Sheldrick, 2008 ▶); molecular graphics: *SHELXTL*; software used to prepare material for publication: *SHELXTL*.

## Supplementary Material

Crystal structure: contains datablocks global, I. DOI: 10.1107/S160053680904923X/kp2235sup1.cif

Structure factors: contains datablocks I. DOI: 10.1107/S160053680904923X/kp2235Isup2.hkl

Additional supplementary materials:  crystallographic information; 3D view; checkCIF report

## Figures and Tables

**Table 1:** Selected bond lengths (Å)

Cu1—Cl3	2.2183 (9)
Cu1—Cl1	2.2333 (8)
Cu1—Cl2	2.2426 (9)
Cu1—Cl4	2.2517 (9)

**Table 2:** Hydrogen-bond geometry (Å, °)

*D*—H⋯*A*	*D*—H	H⋯*A*	*D*⋯*A*	*D*—H⋯*A*
N1—H1A⋯Cl4	0.86	2.93	3.453 (4)	121
N1—H1A⋯Cl2	0.86	2.95	3.655 (4)	141
N1—H1B⋯Cl3^i^	0.86	2.60	3.399 (4)	157
N1—H1B⋯Cl1^i^	0.86	2.95	3.511 (4)	125
N3—H3B⋯Cl1^ii^	0.86	2.51	3.347 (4)	166
N3—H3B⋯Cl2^ii^	0.86	2.86	3.277 (4)	112
N2—H2⋯Cl2	0.86	2.31	3.162 (4)	171
N3—H3A⋯Cl4	0.86	2.85	3.585 (4)	144
N4—H4⋯Cl4	0.86	2.36	3.204 (4)	169
C6—H6C⋯Cl3^iii^	0.96	2.78	3.670 (4)	155
C12—H12B⋯Cl1^iv^	0.96	2.94	3.781 (4)	147

Symmetry codes: (i) 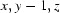; (ii) 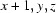; (iii) 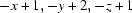; (iv) 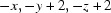.

**Fig. 1.**
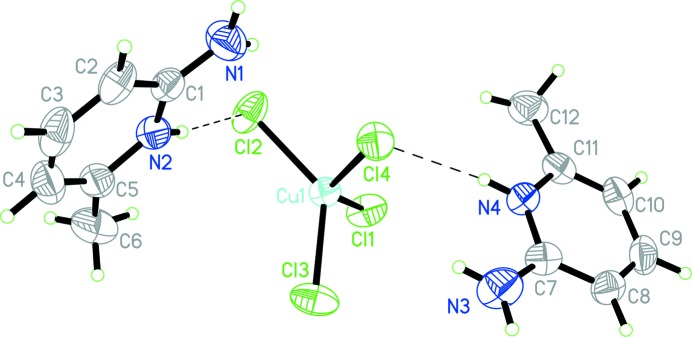
The molecular structure of (I).

**Fig. 2.**
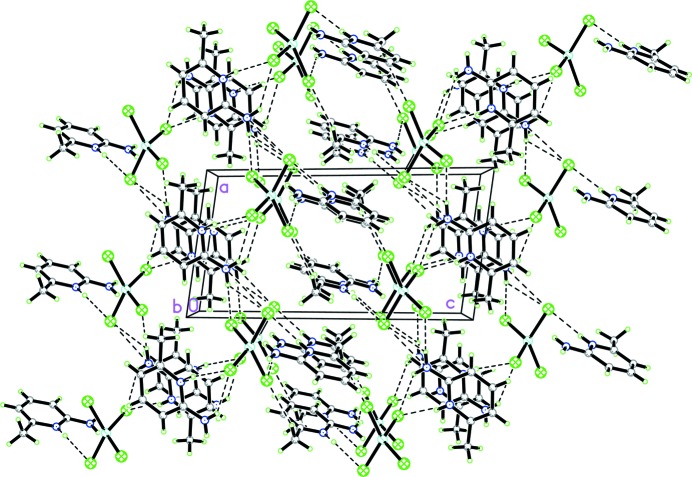
A packing diagram viewed down along the b axis. Hydrogen bonds are illustrated as thin lines.

## supplementary crystallographic information

### Comment

There are a series of compounds of the formula A_2_[*MX*_4_], where A is an organic cation, usually a protonted base, *M* is a divalent transition metal ion and *X* is a halide (Cl, Br) (Hammar *et al.*, 1997). A wide variety of these complexes are known where the A-group is a protonated alkylamine (Zhou *et al.*, 1990), heterocycle such as pyridine (Place *et al.*, 1987), 2-aminopyrimidine (Zanchini *et al.*, 1990), or 2-amino-3-methylpridine (Coffey *et al.*, 2000). The crystal structure of the title compound, (I), is now subjected to X-ray structure analysis.

The asymmetric unit comprises the two protonated, 2-amino-6-methyl-pyridinium cations (HAMP) and [CuCl_4_]^2-^ anion (Fig. 1, Table1). The dihedral angle of the two HAMP cations is of 97.0 (3)°. The [CuCl_4_]^2-^ anion assumes a distorted tetrahedral geometry consistent with the anticipated by Jahn–Teller effect documented by the value of the *trans* Cl—Cu—Cl angle and also by the dihedral angle between CuCl_2_ planes. In the present structure, the two independent *trans* angles are 132.57 (4)° and 129.70 (4)° and the dihedral angle between the CuCl_2_ planes is 65.4°. The average value of 2.2365Å observed in the [CuCl_4_]^2-^ anion is shorter than the average value of 2.270Å (Antolini *et al.*, 1988) or 2.260Å (Zhang *et al.*, 2005) of square planar [CuCl_4_]^2-^anion. In the cation, the N3—C7 bond [1.330 (4) Å] is shorter than the N4—C7 [1.345 (4) Å] and N4—C11 [1.362 (4) Å] bonds, and the C7—C8 [1.394 (4) Å] and C(9)—C(10) [1.399 (5) Å] bonds are significantly longer than C8—C9 [1.345 (5) Å] and C10—C11 [1.348 (4) Å] bonds, this are similar to those in the HAMP cation of (C_6_H_9_N_2_)[ZnCl_3_(C_6_H_8_N_2_)] (Jin *et al.*, 2005) and (C_6_H_9_N_2_)_2_[Sb_2_Cl_6_O] (Feng *et al.*, 2007). In contrast, in the solid state structure of AMP, the N—C bond out of ring is clearly longer than that in the ring (Nahringbauer *et al.*, 1997). The geometric features of HAMP cation (N1/N2/C1/C6) resemble those observed in other 2-aminopyridinium structures (Luque *et al.*, 1997; Jin *et al.*, 2000; Jin *et al.*, 2001; Jin *et al.*, 2005) that are believed to be involved in amine-imine tautomerism (Inuzuka *et al.*, 1986; Inuzuka *et al.*, 1990; Ishikawa *et al.*, 2002). Similar features are also provided by cation HAMP (N3/N4/C7/C12). The crystal packing is determined by hydrogen bonds (Fig. 2 and Table 2) and π–π stacking interactions. The X1A^···^X1Ai separation (X1A is the centroid of the C1C5 ring, symmetry code: 1 - *x*, 1 - *y*, 1 - *z*) is of 3.635 (4)°, and the X1B^···^X1Bi separation (X1B is the centroid of the C7C11 ring, symmetry code: 1 - *x*, 2 - *y*, 2 - *z*) is of 3.642 (4)°.

### Experimental

2-Amino-6-methyl-pyridine, aqueous HCl and CuCl_2_^.^2H_2_O in a molar ratio of 2:2:1 were mixed and dissolved in sufficient water. It was kept stirring and heating till a clear solution was obtained. Crystals of (I) were formed by gradual evaporation of excess water over one week at 293 K. Analysis for (I) (%): C 34.06; H 4.25; N, 13.27; Found (%): C 34.02; H 4.28; N 13.22. IR Spectrum (KBr, cm^-1^): 3411 (*s*), 3295 (*s*), 3195 (*m*), 3090 (*m*), 1656 (*versus*), 1630 (w), 1565 (w), 1474 (w), 1392 (*m*), 1309 (*m*), 1174 (w), 1042 (w), 997 (w), 793 (*m*), 715 (w), 612 (w), 564 (w), 421 (*m*).

### Refinement

All the H atoms were placed in calculated positions and allowed to ride on their parent atoms at distances of 0.93 Å for aromatic group, 0.86 Å for amido and 0.96 Å for methyl with isotropic displacement parameters 1.2 times *U*_eq_ of the parent atoms.

### Crystal data

**Table:**

(C_6_H_9_N_2_)_2_[CuCl_4_]	*Z* = 2
*M**_r_* = 423.65	*F*(000) = 430.0
Triclinic, *P*1	*D*_x_ = 1.553 Mg m^−^^3^
Hall symbol: -P 1	Mo *K*α radiation, λ = 0.71073 Å
*a* = 7.7466 (17) Å	Cell parameters from 2451 reflections
*b* = 8.0372 (18) Å	θ = 2.2–24.3°
*c* = 14.969 (3) Å	µ = 1.79 mm^−^^1^
α = 78.922 (4)°	*T* = 273 K
β = 82.154 (4)°	Prism, blue
γ = 89.911 (4)°	0.35 × 0.34 × 0.30 mm
*V* = 905.8 (3) Å^3^	

### Data collection

**Table:**

Bruker SMART APEX area-detector diffractometer	3206 independent reflections
Radiation source: fine-focus sealed tube	3161 reflections with *I* > 2σ(*I*)
graphite	*R*_int_ = 0.013
φ and ω scan	θ_max_ = 25.0°, θ_min_ = 2.6°
Absorption correction: multi-scan (*SADABS*; Bruker, 2000)	*h* = −7→9
*T*_min_ = 0.549, *T*_max_ = 0.578	*k* = −9→9
4783 measured reflections	*l* = −12→17

### Refinement

**Table:**

Refinement on f^2^	Primary atom site location: structure-invariant direct methods
Least-squares matrix: full	Secondary atom site location: difference Fourier map
*R*[*F*^2^ > 2σ(*F*^2^)] = 0.034	Hydrogen site location: inferred from neighbouring sites
*wR*(*F*^2^) = 0.090	H-atom parameters constrained
*S* = 1.05	*w* = 1/[σ^2^(*F*_o_^2^) + (0.0481*P*)^2^ + 0.406*P*] where *P* = (*F*_o_^2^ + 2*F*_c_^2^)/3
3161 reflections	(Δ/σ)_max_ = 0.001
190 parameters	Δρ_max_ = 0.55 e Å^−^^3^
0 restraints	Δρ_min_ = −0.33 e Å^−^^3^

### Special details

**Table:**

Geometry. all e.s.d.'s (except the e.s.d. in the dihedral angle between two l.s. planes) are estimated using the full covariance matrix. the cell e.s.d.'s are taken into account individually in the estimation of e.s.d.'s in distances, angles and torsion angles; correlations between e.s.d.'s in cell parameters are only used when they are defined by crystal symmetry. an approximate (isotropic) treatment of cell e.s.d.'s is used for estimating e.s.d.'s involving l.s. planes.
Refinement. refinement of f^2^ against all reflections. the weighted r-factor wr and goodness of fit s are based on f^2^, conventional r-factors r are based on f, with f set to zero for negative f^2^. the threshold expression of f^2^ > σ(f^2^) is used only for calculating r-factors(gt) *etc*. and is not relevant to the choice of reflections for refinement. r-factors based on f^2^ are statistically about twice as large as those based on f, and r- factors based on all data will be even larger.

### Fractional atomic coordinates and isotropic or equivalent isotropic displacement parameters (Å^2^)

**Table:**

	*x*	*y*	*z*	*U*_iso_*/*U*_eq_	
cu1	0.18139 (4)	0.80739 (4)	0.75385 (2)	0.04615 (13)	
cl4	0.31507 (11)	0.56448 (9)	0.80270 (5)	0.0630 (2)	
cl1	0.04761 (10)	0.98881 (10)	0.83451 (6)	0.0677 (2)	
cl3	0.41383 (10)	0.96162 (10)	0.68092 (7)	0.0757 (3)	
cl2	−0.04646 (10)	0.69927 (13)	0.70434 (6)	0.0734 (3)	
n4	0.4134 (3)	0.7120 (3)	0.97401 (16)	0.0498 (5)	
h4	0.3725	0.6715	0.9318	0.060*	
c8	0.6468 (4)	0.8324 (4)	1.0262 (2)	0.0575 (7)	
h8	0.7613	0.8733	1.0172	0.069*	
c1	0.2333 (4)	0.4070 (3)	0.56633 (19)	0.0506 (6)	
n2	0.1895 (3)	0.5691 (3)	0.54343 (16)	0.0506 (5)	
h2	0.1328	0.6149	0.5855	0.061*	
c11	0.3066 (4)	0.7100 (4)	1.0545 (2)	0.0532 (7)	
c9	0.5450 (5)	0.8298 (4)	1.1068 (2)	0.0646 (8)	
h9	0.5906	0.8679	1.1538	0.077*	
c10	0.3717 (4)	0.7707 (4)	1.1212 (2)	0.0641 (8)	
h10	0.3017	0.7733	1.1766	0.077*	
c5	0.2289 (4)	0.6664 (4)	0.4578 (2)	0.0574 (7)	
n1	0.1881 (4)	0.3267 (3)	0.65263 (19)	0.0753 (8)	
h1a	0.1321	0.3790	0.6922	0.090*	
h1b	0.2148	0.2224	0.6690	0.090*	
c2	0.3220 (4)	0.3304 (4)	0.4975 (2)	0.0610 (8)	
h2a	0.3539	0.2177	0.5104	0.073*	
c3	0.3598 (4)	0.4236 (5)	0.4123 (2)	0.0720 (10)	
h3	0.4166	0.3735	0.3658	0.086*	
c12	0.1255 (4)	0.6423 (5)	1.0599 (3)	0.0741 (9)	
h12a	0.1120	0.6067	1.0035	0.111*	
h12b	0.0440	0.7294	1.0696	0.111*	
h12c	0.1038	0.5474	1.1102	0.111*	
n3	0.6670 (4)	0.7728 (4)	0.8737 (2)	0.0784 (8)	
h3a	0.6183	0.7342	0.8333	0.094*	
h3b	0.7728	0.8112	0.8607	0.094*	
c4	0.3162 (4)	0.5927 (5)	0.3920 (2)	0.0704 (9)	
h4a	0.3471	0.6554	0.3329	0.084*	
c6	0.1734 (5)	0.8456 (4)	0.4464 (3)	0.0841 (11)	
h6a	0.1139	0.8658	0.5037	0.126*	
h6b	0.0965	0.8668	0.4005	0.126*	
h6c	0.2741	0.9200	0.4274	0.126*	
c7	0.5786 (3)	0.7731 (3)	0.9564 (2)	0.0513 (6)	

### Atomic displacement parameters (Å^2^)

**Table:**

	*U*^11^	*U*^22^	*U*^33^	*U*^12^	*U*^13^	*U*^23^
cu1	0.04167 (19)	0.0479 (2)	0.0488 (2)	0.00113 (13)	0.00015 (14)	−0.01383 (14)
cl4	0.0799 (5)	0.0468 (4)	0.0649 (5)	0.0114 (3)	−0.0143 (4)	−0.0143 (3)
cl1	0.0553 (4)	0.0672 (5)	0.0843 (6)	−0.0018 (3)	0.0113 (4)	−0.0387 (4)
cl3	0.0564 (4)	0.0628 (5)	0.0938 (6)	−0.0028 (3)	0.0196 (4)	−0.0012 (4)
cl2	0.0468 (4)	0.1114 (7)	0.0754 (5)	−0.0016 (4)	−0.0041 (4)	−0.0547 (5)
n4	0.0454 (12)	0.0522 (13)	0.0532 (13)	0.0017 (10)	−0.0052 (10)	−0.0151 (10)
c8	0.0479 (15)	0.0499 (15)	0.075 (2)	0.0064 (12)	−0.0217 (15)	−0.0052 (14)
c1	0.0541 (15)	0.0491 (15)	0.0523 (16)	−0.0018 (12)	−0.0095 (12)	−0.0177 (12)
n2	0.0494 (13)	0.0517 (13)	0.0511 (13)	−0.0010 (10)	0.0017 (10)	−0.0171 (10)
c11	0.0483 (15)	0.0512 (15)	0.0540 (16)	0.0060 (12)	−0.0003 (13)	0.0003 (13)
c9	0.078 (2)	0.0630 (19)	0.0567 (18)	0.0104 (16)	−0.0305 (17)	−0.0069 (15)
c10	0.071 (2)	0.074 (2)	0.0435 (15)	0.0107 (16)	−0.0065 (14)	−0.0028 (14)
c5	0.0474 (15)	0.0672 (18)	0.0556 (17)	−0.0057 (13)	−0.0081 (13)	−0.0059 (14)
n1	0.106 (2)	0.0559 (15)	0.0605 (17)	0.0022 (14)	−0.0031 (15)	−0.0076 (13)
c2	0.0581 (17)	0.0664 (18)	0.071 (2)	0.0108 (14)	−0.0178 (15)	−0.0373 (16)
c3	0.0570 (18)	0.110 (3)	0.060 (2)	0.0061 (18)	−0.0058 (15)	−0.045 (2)
c12	0.0543 (18)	0.081 (2)	0.079 (2)	−0.0058 (16)	0.0037 (16)	−0.0056 (18)
n3	0.0528 (15)	0.102 (2)	0.082 (2)	−0.0050 (14)	0.0126 (14)	−0.0362 (17)
c4	0.0624 (19)	0.100 (3)	0.0455 (17)	−0.0032 (18)	−0.0046 (14)	−0.0077 (17)
c6	0.078 (2)	0.065 (2)	0.097 (3)	−0.0018 (18)	−0.004 (2)	0.007 (2)
c7	0.0410 (14)	0.0475 (14)	0.0647 (18)	0.0082 (11)	−0.0025 (13)	−0.0124 (13)

### Geometric parameters (Å, °)

**Table:**

Cu1—Cl3	2.2183 (9)	c10—h10	0.9300
Cu1—Cl1	2.2333 (8)	c5—c4	1.348 (5)
Cu1—Cl2	2.2426 (9)	c5—c6	1.488 (5)
Cu1—Cl4	2.2517 (9)	n1—h1a	0.8600
N4—C7	1.345 (4)	n1—h1b	0.8600
N4—C11	1.362 (4)	c2—c3	1.343 (5)
n4—h4	0.8600	c2—h2a	0.9300
c8—c9	1.345 (5)	c3—c4	1.386 (5)
c8—c7	1.394 (4)	c3—h3	0.9300
c8—h8	0.9300	c12—h12a	0.9600
C1—N1	1.328 (4)	c12—h12b	0.9600
C1—N2	1.337 (4)	c12—h12c	0.9600
c1—c2	1.401 (4)	N3—C7	1.330 (4)
N2—C5	1.363 (4)	n3—h3a	0.8600
n2—h2	0.8600	n3—h3b	0.8600
c11—c10	1.348 (4)	c4—h4a	0.9300
c11—c12	1.492 (4)	c6—h6a	0.9600
c9—c10	1.399 (5)	c6—h6b	0.9600
c9—h9	0.9300	c6—h6c	0.9600
			
cl3—cu1—cl1	100.96 (4)	c1—n1—h1a	120.0
cl3—cu1—cl2	132.57 (4)	c1—n1—h1b	120.0
cl1—cu1—cl2	100.71 (3)	h1a—n1—h1b	120.0
cl3—cu1—cl4	98.61 (4)	c3—c2—c1	118.4 (3)
cl1—cu1—cl4	129.70 (4)	c3—c2—h2a	120.8
cl2—cu1—cl4	99.05 (4)	c1—c2—h2a	120.8
c7—n4—c11	124.2 (3)	c2—c3—c4	121.6 (3)
c7—n4—h4	117.9	c2—c3—h3	119.2
c11—n4—h4	117.9	c4—c3—h3	119.2
c9—c8—c7	119.2 (3)	c11—c12—h12a	109.5
c9—c8—h8	120.4	c11—c12—h12b	109.5
c7—c8—h8	120.4	h12a—c12—h12b	109.5
n1—c1—n2	118.3 (3)	c11—c12—h12c	109.5
n1—c1—c2	123.6 (3)	h12a—c12—h12c	109.5
n2—c1—c2	118.1 (3)	h12b—c12—h12c	109.5
c1—n2—c5	124.3 (2)	c7—n3—h3a	120.0
c1—n2—h2	117.8	c7—n3—h3b	120.0
c5—n2—h2	117.8	h3a—n3—h3b	120.0
c10—c11—n4	117.9 (3)	c5—c4—c3	120.1 (3)
c10—c11—c12	125.8 (3)	c5—c4—h4a	119.9
n4—c11—c12	116.4 (3)	c3—c4—h4a	119.9
c8—c9—c10	121.2 (3)	c5—c6—h6a	109.5
c8—c9—h9	119.4	c5—c6—h6b	109.5
c10—c9—h9	119.4	h6a—c6—h6b	109.5
c11—c10—c9	119.7 (3)	c5—c6—h6c	109.5
c11—c10—h10	120.2	h6a—c6—h6c	109.5
c9—c10—h10	120.2	h6b—c6—h6c	109.5
c4—c5—n2	117.3 (3)	n3—c7—n4	118.2 (3)
c4—c5—c6	126.2 (3)	n3—c7—c8	124.0 (3)
n2—c5—c6	116.4 (3)	n4—c7—c8	117.8 (3)
			
n1—c1—n2—c5	179.5 (3)	n1—c1—c2—c3	179.5 (3)
c2—c1—n2—c5	−1.6 (4)	n2—c1—c2—c3	0.5 (4)
c7—n4—c11—c10	−1.1 (4)	c1—c2—c3—c4	1.2 (5)
c7—n4—c11—c12	178.0 (3)	n2—c5—c4—c3	1.0 (5)
c7—c8—c9—c10	−0.8 (4)	c6—c5—c4—c3	−180.0 (3)
n4—c11—c10—c9	−1.3 (4)	c2—c3—c4—c5	−2.1 (5)
c12—c11—c10—c9	179.8 (3)	c11—n4—c7—n3	−177.7 (3)
c8—c9—c10—c11	2.2 (5)	c11—n4—c7—c8	2.5 (4)
c1—n2—c5—c4	0.8 (4)	c9—c8—c7—n3	178.8 (3)
c1—n2—c5—c6	−178.3 (3)	c9—c8—c7—n4	−1.4 (4)

### Hydrogen-bond geometry (Å, °)

**Table:**

*D*—H···*A*	*D*—H	H···*A*	*D*···*A*	*D*—H···*A*
N1—H1A···Cl4	0.86	2.93	3.453 (4)	121.4
N1—H1A···Cl2	0.86	2.95	3.655 (4)	141.1
N1—H1B···Cl3^i^	0.86	2.60	3.399 (4)	156.6
N1—H1B···Cl1^i^	0.86	2.95	3.511 (4)	125.0
N3—H3B···Cl1^ii^	0.86	2.51	3.347 (4)	165.5
N3—H3B···Cl2^ii^	0.86	2.86	3.277 (4)	112.0
N2—H2···Cl2	0.86	2.31	3.162 (4)	171.0
N3—H3A···Cl4	0.86	2.85	3.585 (4)	144.3
N4—H4···Cl4	0.86	2.36	3.204 (4)	169.3
C6—H6C···Cl3^iii^	0.96	2.78	3.670 (4)	154.7
C12—H12B···Cl1^iv^	0.96	2.94	3.781 (4)	146.5

Symmetry codes: (i) *x*, *y*−1, *z*; (ii) *x*+1, *y*, *z*; (iii) −*x*+1, −*y*+2, −*z*+1; (iv) −*x*, −*y*+2, −*z*+2.
